# Empagliflozin’s role in early tubular protection for type 2 diabetes patients

**DOI:** 10.1186/s10020-024-00881-0

**Published:** 2024-07-31

**Authors:** Chuangbiao Zhang, Weiwei Ren, Xiaohua Lu, Lie Feng, Jiaying Li, Beibei Zhu

**Affiliations:** 1 Department of Endocrinology, First Affiliated Hospital of Jinan University, No. 613 West Huangpu Avenue, Tianhe District, Guangzhou, 510630 Guangdong Province China; 2 Department of Endocrinology, Guangzhou Baiyun District Maternal And Child Health Hospital, Guangzhou, 51000 Guangdong Province China; 3 Endoscopy Center, First Affiliated Hospital of Jinan University, No. 613 West Huangpu Avenue, Tianhe District, Guangzhou, 510630 Guangdong Province China

**Keywords:** SGLT2 inhibitor, Empagliflozin, Type 2 diabetes, Normoalbuminuria, Tubular injury biomarkers, Renal function

## Abstract

**Background:**

Patients with type 2 diabetes often face early tubular injury, necessitating effective treatment strategies. This study aimed to evaluate the impact of the SGLT2 inhibitor empagliflozin on early tubular injury biomarkers in type 2 diabetes patients with normoalbuminuria.

**Methods:**

A randomized controlled clinical study comprising 54 patients selected based on specific criteria was conducted. Patients were divided into an intervention group (empagliflozin, *n* = 27) and a control group (*n* = 27) and treated for 6 weeks. Tubular injury biomarkers KIM-1 and NGAL were assessed pre- and post-treatment.

**Results:**

Both groups demonstrated comparable baseline characteristics. Post-treatment, fasting and postprandial blood glucose levels decreased similarly in both groups. The intervention group exhibited better improvements in total cholesterol, low-density lipoprotein, and blood uric acid levels. Renal function indicators, including UACR and eGFR, showed greater enhancements in the intervention group. Significant reductions in KIM-1 and NGAL were observed in the intervention group.

**Conclusion:**

Treatment with empagliflozin in type 2 diabetes patients with normoalbuminuria led to a notable decrease in tubular injury biomarkers KIM-1 and NGAL. These findings highlight the potential of SGLT2 inhibitors in early tubular protection, offering a new therapeutic approach.

**Supplementary Information:**

The online version contains supplementary material available at 10.1186/s10020-024-00881-0.

## Introduction

Type 2 diabetes is a global chronic disease. Diabetic kidney disease (DKD) is one of the most common microvascular complications and a major cause of end-stage renal disease (ESRD) (KDOQI, 2007; Guo et al. 2022; Rayego-Mateos et al. 2023; Barrera-Chimal et al. 2022). With the increasing number of diabetes patients, DKD has become one of the leading causes of kidney disease worldwide, significantly impacting the health and quality of life of patients (Ruszkiewicz et al. 2020; Artasensi et al. 2020; Wong et al. 2022). Therefore, early identification and intervention in the development of DKD are crucial for improving patient prognosis.

In the diagnosis and monitoring of DKD, kidney tubular injury biomarkers such as KIM-1 and NGAL play an important role (Baer et al. 2020). These biomarkers can be detected in the early stages of kidney damage and provide important tools for early diagnosis (Zeni et al. 2017; Thipsawat 2021; Opazo-Ríos et al. 2020; Lu et al. 2020). Changes in their levels can help doctors understand the pathological process of kidney disease and provide a basis for more effective treatments.

SGLT2 inhibitors, with empagliflozin as a representative, are new drugs for treating type 2 diabetes (Zhao et al. 2023; Ferreira et al. 2022; Lu et al. 2022). Several large RCT studies, such as EMPA-REG, DECLARE, CANVAS, CREDENCE, and DAPA-CKD, have shown that SGLT2 inhibitors significantly reduce the risk of kidney endpoints, demonstrating renal protective effects (Wanner et al. 2016; Wiviott et al. 2019; Neal et al. 2017; Perkovic et al. 2019; Wheeler et al. 2020; Angelini et al. 2021). Empagliflozin has been widely used in clinical practice for treating patients with type 2 diabetes, especially those who require simultaneous management of blood glucose and cardiovascular risk (Packer et al. 2020; Voors et al. 2022; Zannad et al. 2020).

Although the effects of empagliflozin in controlling blood glucose and improving cardiovascular conditions have been widely recognized, further research is needed to explore its potential role in tubular protection (Rao 2022; Beles et al. 2023; Herat et al. 2020). The “tubulocentric theory” suggests that tubular injury may be the “driving force” behind the occurrence and development of DKD, even in the early stages (Vergnaud et al. 2023; Lai et al. 2022; Kale et al. 2023; Hendy et al. 2023). Empagliflozin may potentially provide early renal protection by improving tubulointerstitial lesions. However, clinical studies are required to further validate this potential (John et al. 2022; Tariq et al. 2022; Islam et al. 2022).

This study aims to evaluate, for the first time, the impact of empagliflozin on tubular injury markers in early low-risk type 2 diabetes patients with normoalbuminuria. It is the first study to include exclusively early low-risk type 2 diabetes patients with normoalbuminuria, assessing the early tubular protective effects of SGLT2 inhibitors and evaluating the dual regulatory effects of empagliflozin on blood glucose and lipids. By analyzing the changes in tubular injury markers (KIM-1, NGAL) before and after treatment, this study is expected to offer new insights into empagliflozin’s role in early renal protection. These findings are crucial for understanding the potential of SGLT2 inhibitors in mitigating early tubular injury and may provide novel perspectives for future treatment strategies and patient management.

## Materials and methods

### Study objectives

The present study adopts a randomized controlled clinical trial design, strictly adhering to the principles of the Helsinki Declaration and standards of clinical trial research. Prior to the inclusion of each participant, research personnel systematically introduce the study’s objectives, procedures, and potential risks. Written informed consent is obtained from each participant before the commencement of the study. The research protocol has been reviewed and approved by the Ethics Committee of the First Affiliated Hospital of Jinan University.

In this randomized controlled clinical trial targeting patients with type 2 diabetes, the study participants underwent at least 12 weeks of stable baseline treatment before enrollment, ensuring the homogeneity and reliability of the study. This baseline treatment comprised three aspects. Firstly, diabetes education was provided to emphasize the importance of a diabetes diet, regular exercise, regular blood glucose monitoring, and consistent medication adherence to ensure the self-management capacity of the patients throughout the entire study period. Secondly, based on the patient’s blood glucose levels, an appropriate hypoglycemic regimen was selected, consisting of a stable dose of a single medication or a combination of two medications(Górriz et al. 2015) (including insulin, metformin, α-glucosidase inhibitors, or insulin secretagogues, but excluding hypoglycemic medications that may have independent renal protective effects such as empagliflozin, thiazolidinediones, GLP-1 receptor agonists, DPP-4 inhibitors, or SGLT2 inhibitors). The target for blood glucose control was a fasting blood glucose level between 4.4 mmol/L and 7.0 mmol/L and a postprandial blood glucose level of ≤ 10.0 mmol/L after 2 h. For at least 4 weeks prior to enrollment, the type and dose of oral hypoglycemic drugs were kept unchanged, and if the patient was using insulin, the total insulin dose was adjusted by ≤ 10%. Lastly, for patients with comorbid hypertension and dyslipidemia, dietary guidance was provided, and antihypertensive and lipid-lowering drugs were used for treatment. ACE inhibitors or ARBs were the preferred options for blood pressure control, and if blood pressure control was inadequate despite using the maximum tolerated dose, other antihypertensive drugs, except diuretics, were added. For patients with dyslipidemia, dietary guidance or combination therapy using lipid-lowering drugs was implemented, with statins used for elevated cholesterol levels and fibrates for elevated triglyceride levels, and both drugs used if necessary. The blood pressure and lipid control targets were based on the 2017 edition of “Guidelines for the Prevention and Treatment of Type 2 Diabetes Mellitus in China”. For at least 4 weeks prior to enrollment, the aforementioned types and doses of oral medication remained unchanged.

Through this study, we hope to further explore the potential of SGLT2 inhibitors in early tubular protection, providing scientific support and new treatment strategies for the early prevention and treatment of DKD.

### Inclusion and exclusion criteria

Screening will be conducted on type 2 diabetes patients who received basic treatment at the outpatient or inpatient department of The First Affiliated Hospital of Jinan University from July 2019 to July 2023.

#### Inclusion criteria

(1) Glycated hemoglobin level between 6.5% and 9.0%. (2) UACR less than 30 mg/g and eGFR equal to or greater than 60 ml/min/1.73 m^2^. (3) Meeting the diagnostic criteria for diabetes according to the 1999 World Health Organization (WHO) guidelines. (4) Age above 18 and below 70 years. (5) No alcohol or drug dependency, no severe psychiatric or intellectual impairments. (6) Obtaining informed consent and cooperation from the patient and their family, with the signing of an informed consent form.

#### Exclusion criteria

(1) Significant increase in fasting blood glucose (≥ 11.1 mmol/L) or substantial rise in blood pressure (≥ 180/110 mmHg) after initial treatment, with evident clinical symptoms of hyperglycemia or hypertension. (2) Severe hyperlipidemia (low-density lipoprotein cholesterol ≥ 4.9 mmol/L or total cholesterol ≥ 7.2 mmol/L or triglycerides ≥ 5.6 mmol/L) or severe hyperuricemia (serum uric acid ≥ 540 µmol/L) following initial treatment. (3) If ACE inhibitors (ACEI) or angiotensin receptor blockers (ARB) were not used during initial treatment, subsequent use may be required due to the patient’s condition. (4) Use of corticosteroids or other medications that significantly affect blood glucose levels, or the necessity of using drugs such as prednisone or diuretics that affect renal metabolism due to the patient’s condition. (5) Recent occurrence of acute cardiovascular or cerebrovascular events within the past 3 months, major gastrointestinal surgeries within the past 2 years, or a history of cancer within the past 5 years. (6) Coexistence of primary or secondary renal diseases, such as gouty nephropathy, kidney stones, renal cysts, renal transplantation, urinary tract infections, etc. (7) Presence of severe infections, diabetic ketoacidosis, diabetic ketoacidosis coma, or hyperosmolar hyperglycemic state. (8) Severe organic abnormalities in the heart, liver, kidney, brain, or other organs. (9) Pregnant or breastfeeding women. (10) Type 1 diabetes and other specific types of diabetes.

#### Criteria for withdrawal

(1) Withdrawal must be initiated if there is a significant change in the patient’s condition that requires urgent intervention and affects the continuity of the study. (2) Withdrawal should be considered if the patient develops severe complications such as severe infection, ketoacidosis, hyperosmolar non-ketotic diabetic coma, or significant organ dysfunction (e.g., heart, liver, brain) during the treatment period. (3) If the participant experiences serious adverse events or reactions, such as ketoacidosis or genitourinary tract infections, withdrawal from the clinical trial is necessary. (4) Withdrawal may be necessary if the participant requires treatment for other illnesses during the study, which may interfere with the trial.

#### Exclusion criteria

(1) Participants who fail to adhere to the prescribed medication regimen and significantly deviate from the trial protocol. (2) Participants with incomplete clinical data. (3) Participants who voluntarily withdraw from the clinical trial. (4) Participants who are unable to cooperate with follow-up procedures.

### Randomization and drug intervention in group assignment

Patients who meet the inclusion criteria will be randomly assigned into two groups, the control group and the intervention group (empagliflozin group), using a random number table. The randomization process will be conducted by experienced professionals who will not be involved in the follow-up and statistical analysis of the patients.

After randomization, drug treatment will be implemented as follows:

1. Intervention Group (Empagliflozin Group): In addition to the standard treatment, the patients in this group will receive empagliflozin, an SGLT2 inhibitor, also known as empagliflozin tablets (trade name: Jardiance/Ou Tangjing, Shanghai Boehringer Ingelheim Pharmaceuticals Ltd., Drug Registration Certificate: H20170351, China Drug Approval Number: J20171073). Empagliflozin will be administered orally at a dose of 10 mg once daily on an empty stomach in the morning. The dose of empagliflozin will remain unchanged, but other antidiabetic drugs (of the same class as the baseline therapy) may be adjusted based on blood glucose levels.
2. Control Group: In the control group, patients will receive the standard treatment without the addition of empagliflozin. Similar to the intervention group, the dose of antidiabetic drugs (of the same class as the baseline therapy) may be adjusted based on blood glucose levels.

The target for glycemic control in both groups is fasting blood glucose between 4.4 mmol/L and 7.0 mmol/L and postprandial blood glucose below 10.0 mmol/L. The maximum recommended dose of oral antidiabetic drugs should not be exceeded, and combining two different types of oral antidiabetic drugs is not recommended. It is also advised to avoid using four or more different types of oral antidiabetic drugs concurrently. The total dose of insulin should not exceed 1 U/kg. During follow-up, the dosage of ACE inhibitors or ARBs should remain unchanged, while other antihypertensive and lipid-lowering regimens should follow the methods and targets of the baseline therapy stage.

### Research method

Prior to the intervention, we recorded basic patient information, including contact details, age, gender, height, weight, BMI, blood pressure, duration of diabetes, history of hypertension, cardiovascular disease history, smoking status, family history, and concurrent medication usage. Before the intervention, we conducted laboratory tests to observe the following indicators: (1) Routine laboratory tests: Fasting blood glucose, lipid profile, blood uric acid, liver function, blood creatinine, blood urea nitrogen, and postprandial blood glucose (measured using an automated biochemical analyzer, Model 7600 Series, Hitachi) under fasting conditions. We also used fresh morning urine samples to measure urinary microalbumin (measured using an automated luminescent system, MAGLUMI 4000, Shenzhen New Industries Biomedical Engineering Co., Ltd.) and urinary creatinine (measured using an automated biochemical analyzer, Model 7600 Series, Hitachi), and calculated the UACR. Furthermore, we used high-performance liquid chromatography (D-10 kit, Bio-Rad, USA) to measure glycated hemoglobin (HbA1c). (2) We calculated the eGFR using the modified diet in renal disease (MDRD) equation for the Chinese population (Ma et al. 2006).

### ELISA detection of IL8, A1M, B2M, L-FABP, KIM-1, and NGAL

The first step involves the collection and storage of urine samples. Fresh morning urine specimens should be collected on an empty stomach, with each sample volume being 10 ml. Immediately after collection, the samples should be centrifuged at 3000 rpm for 10 min at 4℃ to separate sediment from the urine. Following centrifugation, the supernatant should be transferred to EP tubes and stored at -80℃ for subsequent analysis. When performing the experimental measurements, the frozen samples should be thawed and used immediately to avoid the impact of repeated freeze-thaw cycles.

For the ELISA detection of KIM-1, the Human Urinary TIM-1/KIM-1/HAVCR Quantikine ELISA Kit (catalog number DKM100) from R&D Systems, USA, was utilized. As for NGAL, the Human Lipocalin-2/NGAL Quantikine ELISA Kit (catalog number DLCN20) from R&D Systems, USA, was employed. IL-18 was detected using the Human IL-18 ELISA Kit (catalog number EK118–48, Sencken Biotech, China), L-FABP was analyzed using the Human Liver-Type Fatty Acid-Binding Protein (L-FABP) ELISA kit from Wuhan Merck Biomaterials Co., Ltd. (catalog number 69-75621), A1M was measured using the α1-Microglobulin (A1M) detection kit from Wuhan Yunke Long Biotechnology Co., Ltd. (catalog number: SCA217Hu), and B2M was quantified with the β2-Microglobulin (B2M) detection kit from Wuhan Yunke Long Biotechnology Co., Ltd. (catalog number: SEA260Hu). Before conducting the experiment, the reagents need to be prepared. This includes allowing the kit to reach room temperature from the recommended storage temperature of 2–8℃, as well as preparing washing solution, color reagent, diluent, and KIM-1 standards.

The experimental procedure involves retrieving the microwell plate from the sealed aluminum bag and adding assay diluent, standard curve samples, as well as test samples for incubation. This is followed by plate washing and incubation with Human TIM-1 Conjugate. Subsequently, the plate is washed again, and a color reagent is added for the reaction. Finally, a stop solution is added, and the absorbance is measured using an ELISA reader (ELx808, BIOTEK). The concentration of KIM-1 in the samples is calculated based on the standard curve, and then the results are corrected using the urine creatinine ratio to obtain the final KIM-1 or NGAL concentration levels. The detection methods for IL-8, A1M, B2M, and L-FABP were strictly carried out in accordance with the instructions provided by the respective assay kits.

### Follow-up observation of treatment progress

During the treatment period, the patients will be followed up through weekly phone calls to observe their treatment progress. Diabetes education will continue to be provided, emphasizing the importance of medication adherence. Good adherence is defined as the actual medication intake being more than 80% of the prescribed dosage. The patients will be instructed to perform self-monitoring of blood glucose, with a frequency of 3 days per week and 5–7 times per day. Self-monitoring of blood pressure will also be performed, with a frequency of at least 3 times per day for 3 days per week. If necessary, the monitoring frequency will be increased based on the blood glucose and blood pressure levels. The patients will be asked to record their blood glucose and blood pressure levels, and if needed, they will be advised to return to the hospital for treatment adjustment. Adverse reactions during the treatment process will be monitored, and any adverse events, especially ketoacidosis genital and urinary tract infections, will be systematically investigated and recorded. Patients will be reminded to maintain personal hygiene of the external genitalia, drink an appropriate amount of water, and ensure smooth urination.

### Follow-up observational indicators

After 6 weeks of treatment, the patients were readmitted for a follow-up assessment, where the baseline observational indicators were re-examined under the same conditions. The patient’s systolic and diastolic blood pressure were recorded, and fasting blood glucose, blood lipid profile, uric acid levels, hepatic function, blood creatinine, UACR, urinary kidney injury molecule-1 (KIM-1), urinary neutrophil gelatinase-associated lipocalin (NGAL), and postprandial blood glucose at 2 h were measured under fasting conditions. The testing methods used for these indicators remained consistent with those employed prior to the intervention. The primary focus of observation was on urinary KIM-1 and NGAL.

### Statistical analysis

For continuous variable data, if it follows a normal distribution, the mean and standard deviation are used to represent it. If it follows a skewed distribution, the median (interquartile range) is used. For categorical data, frequency (percentage) is used. To compare the data before and after treatment, a repeated-measures analysis of variance is used for the pre-and post-test data in a controlled design. For comparing differences between two groups, the two-sample t-test, the Mann-Whitney U test, and the chi-square test are used for normally distributed data, skewed data, and categorical data, respectively. All statistical analyses are performed using SPSS 19.0 software, and a significance level of *p* < 0.05 is considered statistically significant.

## Results

### Analysis of baseline characteristics in the clinical trial of type 2 diabetes patients

Detailed analysis of baseline characteristics among participants in a clinical trial for the treatment efficacy of type 2 diabetes is of utmost importance. This helps to ensure comparability between the two groups and provides a foundation for further evaluation of outcomes.

**Fig. 1:**
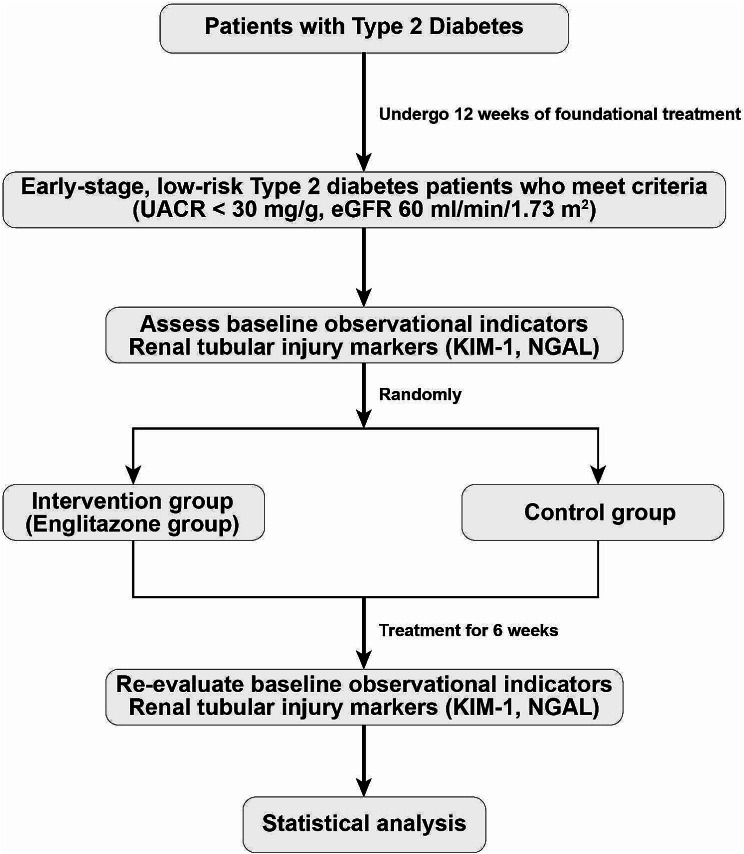
Research technology roadmap

In this study, a total of 54 patients with type 2 diabetes were strictly enrolled and randomized into two groups: the intervention group (empagliflozin group) and the control group, with 27 patients in each group. The specific study procedure is illustrated in Fig. 1. One patient from each group withdrew from the clinical trial due to personal reasons, resulting in no significant difference in overall dropout rates between the two groups. Medication adherence among patients in the intervention group was excellent, with an actual medication intake of more than 80% of the prescribed dose. Both groups demonstrated good glycemic and blood pressure control during the treatment period, and overall general conditions were favorable. No severe adverse events or drug-related reactions were reported.

Table 1 presents the baseline clinical characteristics of two patient groups before receiving treatment. There were no significant differences (*p* > 0.05) between the two groups in terms of gender distribution, age, duration of diabetes, smoking history, body mass index (BMI), glycated hemoglobin (HbA1c) levels, systolic and diastolic blood pressure, UACR, and eGFR. Additionally, there were no statistically significant differences in the prevalence of comorbidities (such as hypertension, coronary heart disease, and stroke) and medication combinations (including metformin, α-glucosidase inhibitors, insulin secretagogues, insulin, RAAS inhibitors, calcium channel blockers, and statins) between the two groups.

**Table 1:** Comparison of basic clinical data of two groups before treatment

Basic Information	Intervention Group (*n* = 27)	Control Group (*n* = 27)	t/x^2^	*p*
Gender (Male/Female)	13/14	14/13	0.07	0.79
Age (years)	58.65 ± 7.29	58.26 ± 6.47	0.21	0.42
Duration of Diabetes (years)	8.96 (5.0-10.75)	7.31 (4.0–10.0)	-	0.34
Smoking History n(%)	4 (14.81)	4 (14.81)	0.15	0.7
BMI (kg/m^2^)	23.66 ± 2.49	23.96 ± 2.26	0.46	0.32
HbA1c (%)	7.63 ± 1.20	7.30 ± 1.51	0.89	0.19
Systolic Blood Pressure (mmHg)	132.43 ± 15.63	127.65 ± 12.74	1.28	0.1
Diastolic Blood Pressure (mmHg)	76.70 ± 9.20	78.15 ± 11.31	0.52	0.3
UACR (mg/g)	20.56 ± 10.22	17.23 ± 12.18	1.09	0.14
eGFR (ml/min/1.73m^2^)	89.92 ± 15.11	88.07 ± 16.89	0.42	0.34
Comorbidities n(%)				
Hypertension	6 (22.22)	5 (18.52)	0.11	0.74
Coronary Heart Disease	2 (7.41)	2 (7.41)	0.27	0.6
Stroke	3 (11.11)	1 (3.7)	0.27	0.6
Medication Combination n(%)				
Metformin	20 (74.07)	22 (81.48)	0.43	0.51
Average metformin dose (mg)	756	821		
α-Glucosidase Inhibitor	16 (59.26)	17 (62.96)	0.08	0.78
Insulin Secretagogues	5 (18.52)	6 (22.22)	0.11	0.74
Insulin	8 (29.63)	7 (25.93)	0.09	0.76
RAAS Inhibitors	6 (22.22)	5 (18.52)	0.27	0.6
β-Blockers	0	0	-	-
Diuretics	0	0	-	-
Calcium Channel Blockers	2 (7.41)	2 (7.41)	0.39	0.58
Statins	22 (81.48)	21 (77.78)	0.11	0.74
Average statin dose (mg)	13.45	13.12		

*Note* RAAS inhibitors, renin-angiotensin-aldosterone system inhibitors

In summary, the baseline characteristics of the intervention and control groups were similar at the start of the clinical trial, indicating good comparability between the two groups. This provides a solid foundation for evaluating treatment efficacy and safety in subsequent assessments. Furthermore, the good adherence and stable condition of patients in both groups during treatment indicate the reliability and effectiveness of the clinical trial.

### Analysis and comparison of clinical metabolic parameters before and after treatment in type 2 diabetes mellitus patients

In the research and treatment of type 2 diabetes mellitus, it is crucial to evaluate the changes in clinical metabolic parameters before and after treatment in order to understand the effectiveness of the therapy. This study aims to assess the efficacy and safety of the intervention by conducting a comprehensive analysis of the changes in parameters such as blood glucose, blood lipids, uric acid (UA), and blood pressure in the intervention group and the control group before and after treatment.

**Fig. 2:**
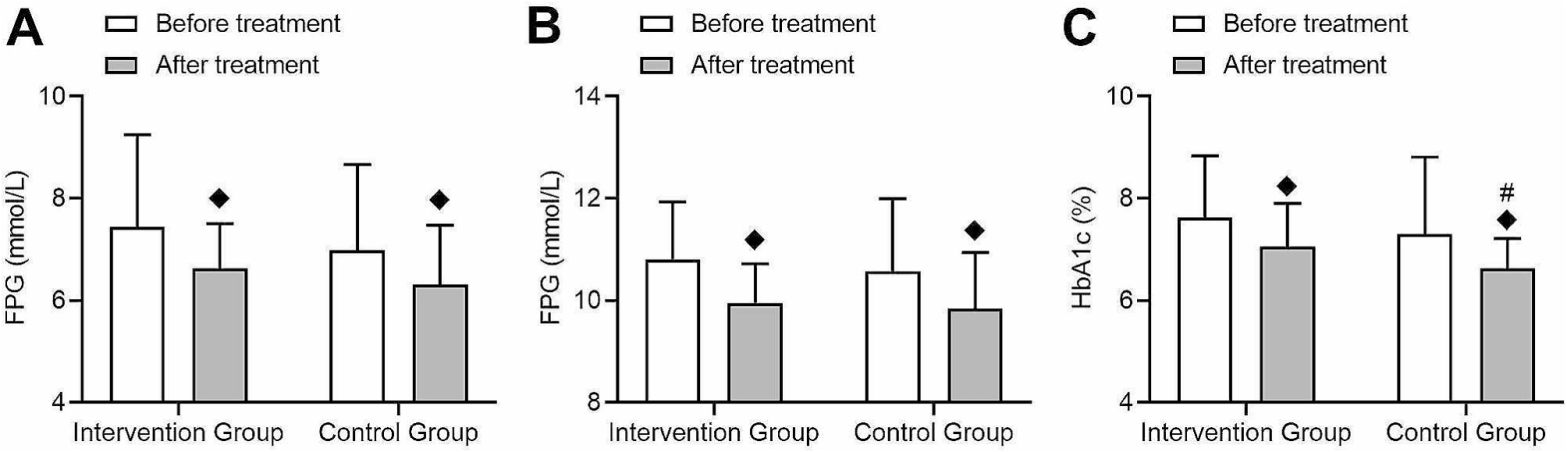
Comparison of changes in blood glucose levels before and after treatment in type 2 diabetes patients. (**A**) Changes in fasting plasma glucose (FPG) levels before and after treatment were measured in the intervention and control groups, with no significant interaction between the two groups. (**B**) Changes in postprandial 2-hour plasma glucose (2hPPG) levels before and after treatment were assessed in the intervention and control groups, with no significant interaction. (**C**) Changes in glycated hemoglobin (HbA1c) levels before and after treatment were examined in the intervention and control groups, with no significant interaction. (◆: Compared to baseline within this group, *p* < 0.05; #: Compared to the post-treatment group, *p* < 0.05)

Table 2 of this study provides detailed records of the changes in clinical metabolic parameters before and after treatment in the intervention group and the control group. Regarding blood glucose control, both the intervention group and the control group showed significant decreases in fasting plasma glucose (FPG) and 2-hour postprandial plasma glucose (2hPPG) after treatment compared to before treatment (intervention group: FPG t = 2.1, *p* = 0.02; 2hPPG t = 3.23, *p* = 0.001; control group: FPG t = 1.7, *p* = 0.04; 2hPPG t = 2.09, *p* = 0.02) (Fig. 2A-B). Additionally, both groups exhibited time effects in FPG and 2hPPG after treatment (FPG F = 5.58, *p* = 0.02; 2hPPG F = 23.68, *p* < 0.01) (Fig. 2A-B), but no significant interaction effects were observed (FPG F = 1.77, *p* = 0.19; 2hPPG F = 0.17, *p* = 0.69) (Fig. 2A-B). The intervention group and the control group both showed significant decreases in glycated hemoglobin (HbA1c) levels after treatment compared to before treatment (intervention group: t = 2.01, *p* = 0.04; control group: FPG t = 2.15, *p* = 0.03). Furthermore, both groups demonstrated a time effect post-treatment (F = 22.96, *p* < 0.01), but no significant interaction effect was observed (F = 0.35, *p* = 0.56) (Fig. 2C).

**Table 2:** Comparison of changes in clinical metabolic indicators before and after treatment

	Intervention Group		Control Group		Inter-group F	*p*	Inter-time F	*p*	Interaction F	*p*
	Before Treatment	After Treatment	Before Treatment	After Treatment						
FPG (mmol/L)	7.44± 1.81	6.63± 0.87^◆^	6.98± 1.68	6.31± 1.17^◆^	0.25	0.62	5.58	**0.02**	1.77	0.19
2hPPG (mmol/L)	10.8± 1.13	9.95± 0.77^◆^	10.57± 1.42	9.85± 1.09^◆^	0.40	0.53	23.68	**0.0**	0.17	0.69
TC (mmol/L)	4.70± 1.24	4.08± 1.05^◆^	4.47± 1.16	4.46± 1.01	0.08	0.78	5.45	**0.02**	5.52	**0.02**
TG (mmol/L)	1.76± 1.07	1.28± 0.52^◆^	1.50± 0.8	1.46± 0.82	0.04	0.84	5.06	**0.03**	3.69	0.06
LDL (mmol/L)	2.67± 0.87	2.11± 0.59^◆^	2.43± 0.74	2.42± 0.73	0.05	0.82	7.15	**0.01**	7.42	**0.01**
HDL (mmol/L)	1.03± 0.26	1.06± 0.31	1.01± 0.19	1.11± 0.20^◆^	0.06	0.82	5.98	**0.02**	1.56	0.22
UA (µmol/L)	379.68± 91.69	314.48± 87.37^◆^	357.25± 106.37	361.61± 90.30^#^	0.31	0.58	4.47	**0.04**	6.38	**0.02**
Systolic Blood Pressure, (mmHg)	132.43± 15.63	123.39± 12.95^◆^	127.65± 12.74	124.3± 14.61	0.86	0.36	2.99	0.09	0.48	0.49
Diastolic Blood Pressure(mmHg)	76.70± 9.20	79.33± 10.76	78.15± 11.31	74.0± 8.82	1.22	0.28	0.19	0.66	1.79	0.19
HbA1c(%)	7.63 ± 1.20	7.06 ± 0.85	7.30 ± 1.51	6.63 ± 0.58 ^#^	0.35	0.56	22.96	0.0	5.79	0.02

*Note* FPG, fasting plasma glucose; 2hPPG, 2-hour postprandial glucose; TC, total cholesterol; TG, triglycerides; LDL, low-density lipoprotein cholesterol; HDL, high-density lipoprotein cholesterol; UA, uric acid; ◆: *p* < 0.05 compared to before treatment in this group, #: *p* < 0.05 compared to Intervention Group, the difference is statistically significant

**Fig. 3:**
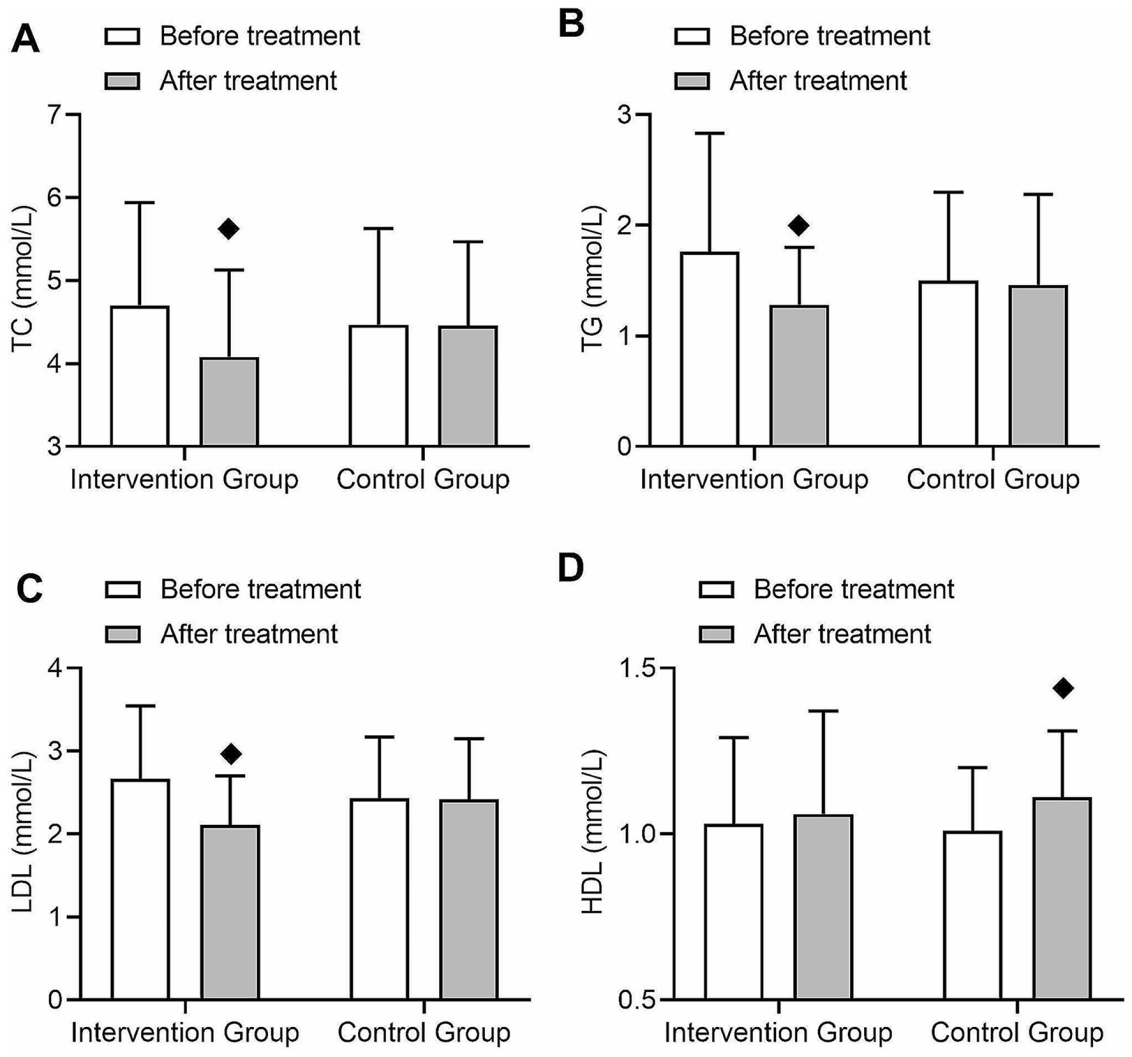
Comparison of changes in blood lipid metabolism indicators before and after treatment in type 2 diabetes patients. (**A**) Changes in total cholesterol (TC) levels before and after treatment in the intervention group and control group, with an interaction effect between the two groups. (**B**) Changes in triglyceride (TG) levels before and after treatment in the intervention group and control group, with an interaction effect between the two groups. (**C**) Changes in low-density lipoprotein (LDL) levels before and after treatment in the intervention group and control group, with an interaction effect between the two groups. (**D**) Changes in high-density lipoprotein (HDL) levels before and after treatment in the intervention group and control group, with an interaction effect between the two groups. (◆: *p* < 0.05 compared to before treatment in this group)

In terms of blood lipid metabolism, the intervention group showed significant reductions in total cholesterol (TC), triglycerides (TG), and low-density lipoprotein (LDL) levels after treatment (TC t = 1.98, *p* = 0.03; TG t = 2.1, *p* = 0.02; LDL t = 2.77, *p* = 0.003), while high-density lipoprotein (HDL) levels did not show significant changes compared to before treatment (Fig. 3A-D). In the control group, there were no significant differences in TC, TG, and LDL levels after treatment compared to before treatment, but HDL levels increased significantly (t = 1.88, *p* = 0.03) (Fig. 3A-D). Both groups showed a time effect in TC, TG, LDL, and HDL levels after treatment (TC F = 5.45, *p* = 0.02; TG F = 5.06, *p* = 0.03; LDL F = 7.15, *p* = 0.01; HDL F = 5.98, *p* = 0.02) (Fig. 3A-D), and significant interactions were observed in TC and LDL levels (TC F = 5.52, *p* = 0.02; LDL F = 7.42, *p* = 0.01) (Fig. 3A-D), indicating a greater improvement in blood lipid metabolism in the intervention group compared to the control group.

**Fig. 4:**
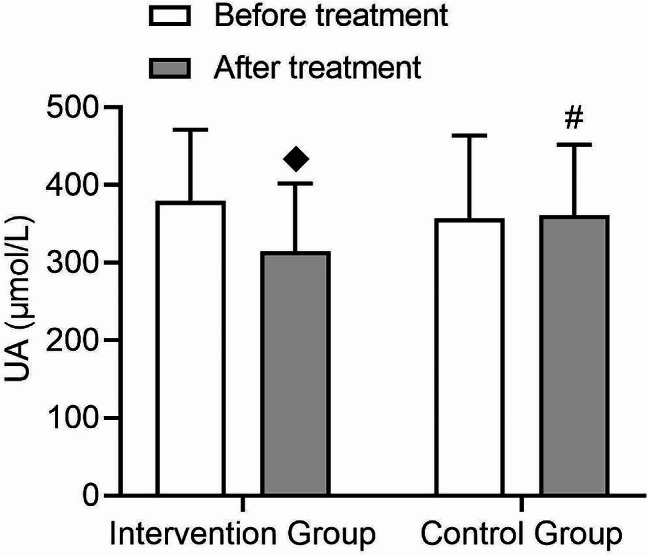
Comparison of changes in uric acid (UA) levels before and after treatment in patients with type 2 diabetes. Changes in uric acid levels before and after treatment in the intervention group and control group, with an interaction effect between the two groups. (◆: *p* < 0.05 compared to before treatment in this group, #: *p* < 0.05 compared after treatment groups)

Analysis of UA showed a significant decrease in UA levels after treatment in the intervention group compared to before treatment (t = 2.68, *p* = 0.01), while there was no significant change in UA levels after treatment in the control group (Fig. 4). Both groups showed a time effect in UA levels after treatment (F = 4.47, *p* = 0.04) (Fig. 4), and a significant interaction was observed (F = 6.38, *p* = 0.02) (Fig. 4), indicating a greater decrease in UA levels in the intervention group.

**Fig. 5:**
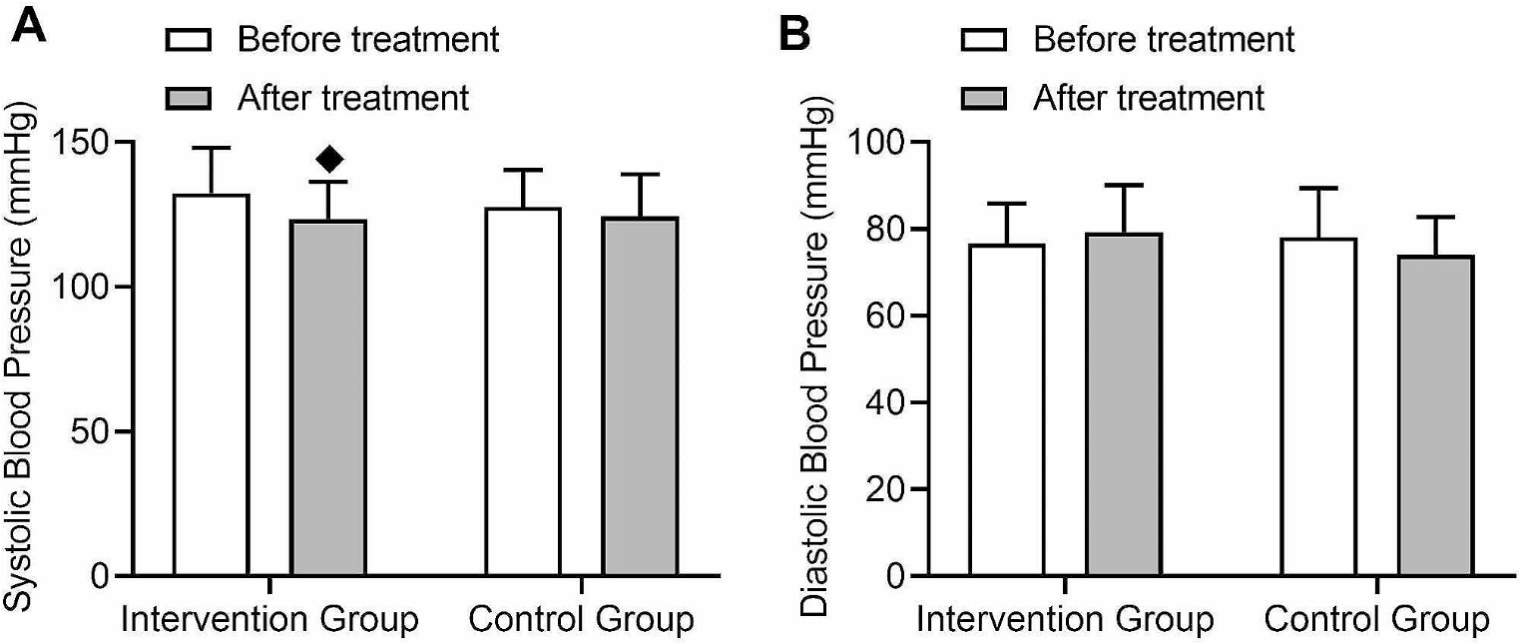
Comparison of changes in blood pressure before and after treatment in patients with type 2 diabetes. (**A**) Changes in systolic blood pressure before and after treatment in the intervention group and control group, with an interaction effect between the two groups. (**B**) Changes in diastolic blood pressure before and after treatment in the intervention group and control group, with an interaction effect between the two groups. (◆: *p* < 0.05 compared to before treatment in this group)

In terms of blood pressure, the intervention group showed a significant decrease in systolic blood pressure after treatment compared to before treatment (t = 2.31, *p* = 0.01) (Fig. 5A), while diastolic blood pressure did not show a significant change (Fig. 5B). The control group did not show significant changes in systolic or diastolic blood pressure after treatment (Fig. 5A-B). There was no significant difference in the magnitude of change in systolic or diastolic blood pressure between the two groups before and after treatment, and no interaction effect was observed.

In summary, both the intervention and control groups showed improvements in clinical metabolic indicators such as blood glucose, blood lipids, uric acid, and blood pressure after treatment, with the intervention group experiencing greater improvements in blood lipid metabolism and uric acid levels compared to the control group. These results suggest that the treatment approach for patients with type 2 diabetes has a significant effect on improving clinical metabolic indicators, particularly in controlling blood lipids and uric acid.

### Comparative analysis of changes in urinary albumin-to-creatinine ratio, glomerular filtration rate, and tubular injury biomarkers in type 2 diabetes patients before and after treatment

**Fig. 6:**
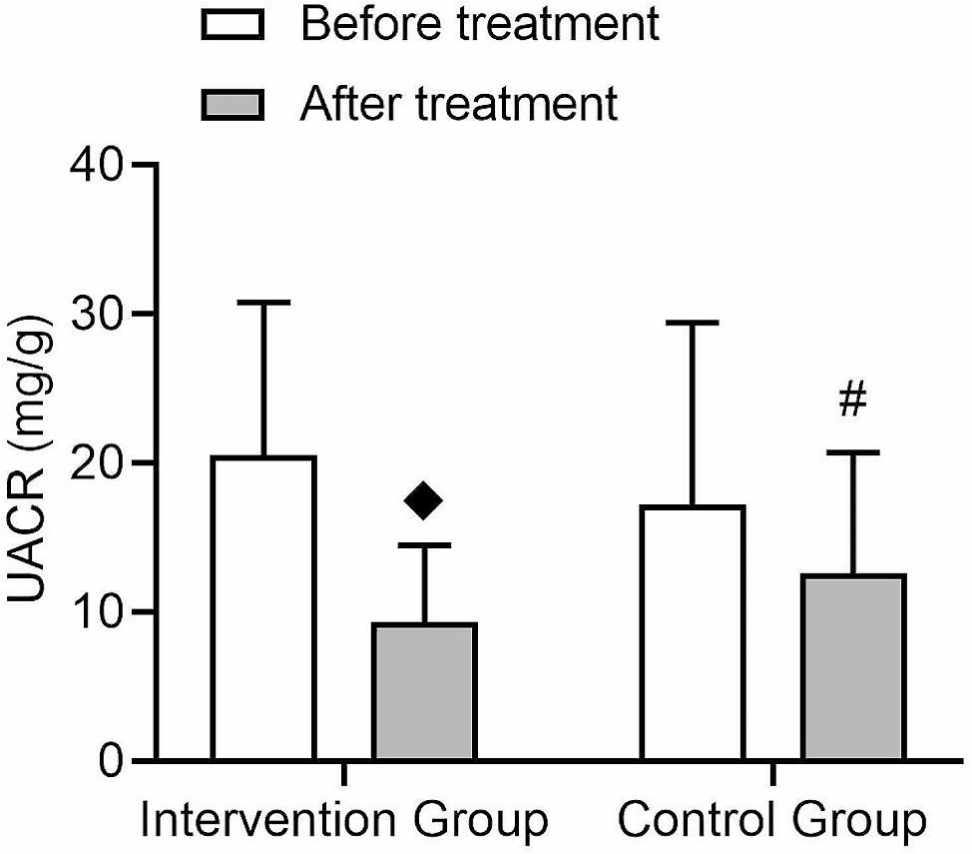
Comparison of changes in UACR before and after treatment in patients with type 2 diabetes. The changes in UACR values before and after treatment were shown between the intervention group and the control group, with an interaction effect present between the two groups. (◆: Compared to before treatment within this group, *p* < 0.05, #: Compared between the post-treatment groups, *p* < 0.05)

The UACR, eGFR, and tubular injury biomarkers such as urinary kidney injury molecule-1/creatinine (UKIM-1/CR) and urinary neutrophil gelatinase-associated lipocalin/creatinine (UNGAL/CR) are important indicators for assessing renal function and damage in the treatment process of type 2 diabetes patients. The research findings revealed that the intervention group showed a significant decrease in UACR after treatment (t = 5.1, *p* = 0.0), while the control group exhibited a decreasing trend but did not reach statistical significance (t = 1.65, *p* = 0.052) (Fig. 6). Both groups demonstrated significant time effects (F = 37.0, *p* = 0.001), with an interaction effect being present (F = 6.51, *p* = 0.01), indicating that the intervention group had a greater improvement in UACR compared to the control group (Table 3). Furthermore, renal tubular injury markers such as IL-8, A1M, B2M, and L-BABP all demonstrated superior improvement in urinary albumin-to-creatinine ratio (UACR) compared to the control group (Table 3).

**Table 3:** Comparison of changes in uacr, egfr, and renal tubular injury markers before and after treatment

	Intervention Group			Control Group			Inter-group F	*p*	Inter-time F	*p*	Interaction F	*p*
	Before Treatment		After Treatment		Before Treatment	After Treatment						
UACR (mg/g)		20.56± 10.22	9.31± 5.17^◆^		17.23± 12.18	12.58± 8.1^#^	0.0	0.98	37.0	**0.001**	6.51	**0.01**
eGFR (ml/min/1.73m^2^)		89.92± 15.11	83.32 ± 12.40^◆^		88.07± 16.89	89.22± 14.07^#^	0.28	0.60	3.58	0.06	8.08	**0.01**
UKIM-1/CR (µg/g)		1.22± 0.62	0.96± 0.33^◆^		1.31± 0.79	1.06± 0.55	0.48	0.49	6.35	**0.02**	0.01	0.97
UNGAL/CR (µg/g)		49.24± 25.6	14.05± 9.19^◆^		37.71± 14.18	44.29± 20.95	2.45	0.12	3.34	0.07	12.42	**0.001**
IL18 (pg/mL)		33.21 ± 2.84	24.26 ± 3.31^◆^		32.16 ± 4.89	25.17 ± 5.87^◆^	0.071	0.79	107.36	0.00	1.26	0.26
A1M (pg/mL)		14.50 ± 4.96	11.41 ± 2.34^◆^		14.68 ± 3.65	12.78 ± 2.65^◆#^	8.29	0.01	26.75	0.00	0.86	0.36
B2M (ng/mL)		104.31 ± 10.64	90.17 ± 15.21^◆^		104.23 ± 7.59	93.37 ± 12.89^◆#^	0.20	0.66	22.72	0.00	1.34	0.25
L-FABP/CR (mg/g)		33.18 ± 5.21	24.59 ± 3.23^◆^		33.10 ± 3.98	25.41 ± 5.64^◆^	0.63	0.43	96.82	0.00	0.09	0.77

*Note* UKIM-1/CR, ratio of urinary KIM-1 to urinary creatinine; UNGAL/CR, ratio of urinary NGAL to urinary creatinine; L-FABP/CR,, ratio of L-fatty acid binding protein to urinary creatinine◆: *p* < 0.05 compared to before treatment in this group, #: *p* < 0.05 compared to Intervention Group, the difference is statistically significant

**Fig. 7:**
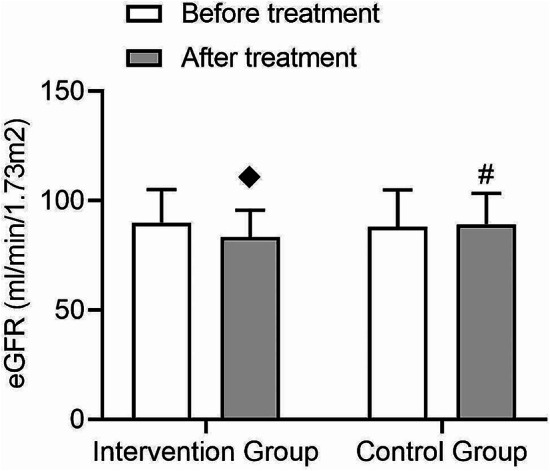
Comparison of changes in eGFR before and after treatment in type 2 diabetes patients. This figure shows the changes in eGFR values before and after treatment in the intervention and control groups, with an interaction effect present between the two groups. (◆: Compared to before treatment within this group, *p* < 0.05, #: Compared between the post-treatment groups, *p* < 0.05)

After treatment, the eGFR in the intervention group showed a slight decrease (t = 1.75, *p* = 0.04), while there was no significant change in the control group (Fig. 7). There was a significant interaction effect between the two groups (F = 8.08, *p* = 0.01), suggesting that the intervention measures may have a specific impact on eGFR.

**Fig. 8:**
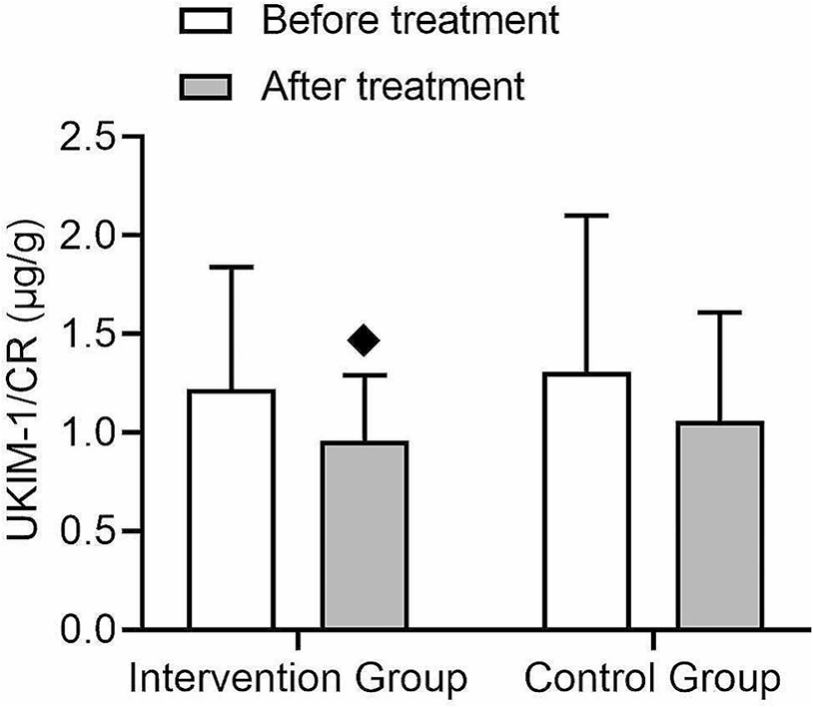
Comparison of changes in urinary kidney injury molecule-1/creatinine (UKIM-1/CR) before and after treatment in type 2 diabetes patients. This figure shows the changes in UKIM-1/CR values before and after treatment in the intervention and control groups, with no interaction between the two groups. (◆: Comparison with the group before treatment *p* < 0.05)

**Fig. 9:**
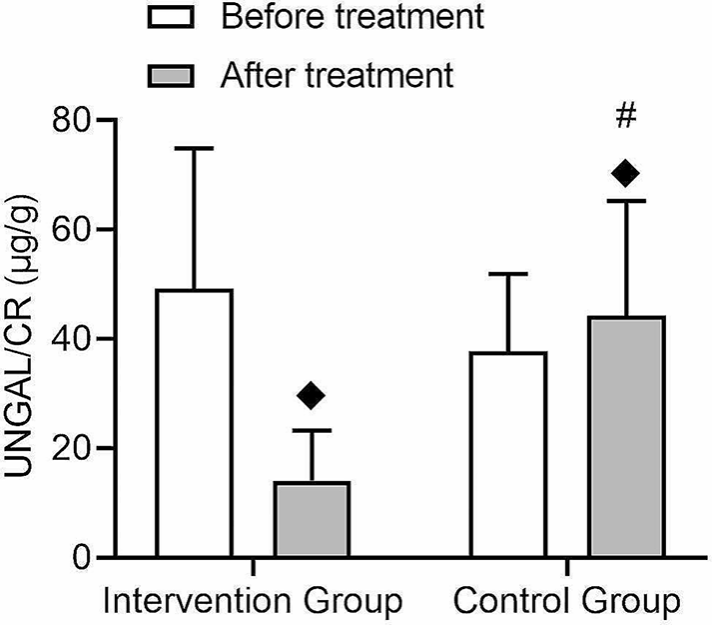
Comparison of changes in urinary neutrophil gelatinase-associated lipocalin/creatinine (UNGAL/CR) before and after treatment in type 2 diabetes patients. This figure shows the changes in UNGAL/CR values before and after treatment in the intervention and control groups, with an interaction between the two groups. (◆: Comparison with the group before treatment *p* < 0.05, #: Comparison between groups after treatment *p* < 0.05)

In the UKIM-1/CR analysis, there was a significant decrease in the intervention group following treatment (t = 1.92, *p* = 0.03), while the control group showed no significant change (Fig. 8). Both groups demonstrated a time effect after treatment (F = 6.35, *p* = 0.02), but no interaction effect was observed (F = 0.01, *p* = 0.97) (Fig. 8). Regarding UNGAL/CR, the intervention group exhibited a significant decrease after treatment (t = 6.72, *p* = 0.0), whereas the control group showed no significant change (Fig. 9). Furthermore, a significant interaction effect was observed (F = 12.42, *p* = 0.001) (Fig. 9). After treatment, a significant difference was observed between the control group and the intervention group in terms of UNGAL/CR and UNGAL/CR, two kidney biomarkers, indicating the protective effect of statin drugs on the kidneys.

In summary, the intervention group displayed more significant improvements in UACR, eGFR, and renal tubular injury markers (especially UNGAL/CR) compared to the control group, indicating the potential benefits of the intervention measures in improving kidney health. However, further research is required to determine the clinical significance of the eGFR decline.

## Discussion

The prevention and treatment of diabetic kidney disease (DKD) has always been a clinical challenge, and there is currently no definitive treatment method for DKD (Limonte et al. 2022; Ma et al. 2023). This study is based on the “renal tubular hypothesis” and explores the impact of SGLT2 inhibitor empagliflozin on renal tubular injury markers in low-risk early-stage type 2 diabetes patients (UACR < 30 mg/g, eGFR ≥ 60 ml/min/1.73 m2), aiming to evaluate its early renal protective effect. Similar to previous studies, we found that after empagliflozin treatment, both fasting blood glucose and postprandial blood glucose significantly decreased in both groups of patients, but there was no significant difference in the degree of glycemic reduction. Furthermore, empagliflozin showed significant effects in lowering total cholesterol and low-density lipoprotein, suggesting its positive impact on lipid metabolism (Lu et al. 2023; Tomita et al. 2020; Xu et al. 2022).

In this study, the significant decrease in serum uric acid levels after empagliflozin treatment is consistent with the known mechanism of SGLT2 inhibitors in reducing uric acid (Chino et al. 2014; Doblado and Moley 2009). In terms of blood pressure, although there was no significant difference compared to the control group, there was a significant decrease in systolic blood pressure within the intervention group, which is related to the diuretic effect of SGLT2 inhibitors to a certain extent (Weber et al. 2016; Kalantar-Zadeh et al. 2021; Burnier and Damianaki 2023; Chen et al. 2023). This study focused on the early renal protection of empagliflozin. In low-risk type 2 diabetes patients included in the study, empagliflozin treatment led to varying degrees of changes in UACR and eGFR. Particularly, the mild decrease in eGFR may be related to the initial use of SGLT2 inhibitors, and this decline is reversible and may be associated with long-term renal protection (Heerspink et al. 2017; Elmore et al. 2014; Kohan et al. 2014; Yoshida 2020; Kjaergaard et al. 2022). This study found that after empagliflozin treatment, the excretion of urinary KIM-1 and NGAL was reduced, indicating the role of empagliflozin in alleviating renal tubular injury. This result emphasizes the renal tubular protective effect of SGLT2 inhibitors, especially their impact on renal tubular injury markers in the early stages. The findings of this study are supported by preclinical research. Lu et al. demonstrated in a diabetic mouse model that empagliflozin prevents iron death by promoting the AMPK-mediated NRF2 activation pathway, thereby alleviating renal tubular damage in diabetic mice (Lu et al. 2023). These findings corroborate the results of this study, indicating the renal protective effect of empagliflozin. Given that the reduction of KIM-1 and NGAL is usually associated with mitigated kidney damage, it can be inferred that the improvement in eGFR may be linked to the decrease in KIM-1 and NGAL levels (Jacobson et al. 2021).

Although this study has made important findings regarding the renal tubular protective effect of SGLT2 inhibitor empagliflozin in early-stage type 2 diabetes patients, there are several limitations. Firstly, the follow-up period of the study is relatively short, which does not fully reveal the long-term effects of empagliflozin treatment on renal tubular injury markers and its sustained effects on patient renal function and overall health status. Secondly, the lack of renal histopathological examination means that the pathological changes in the kidneys cannot be directly confirmed at the histological level, limiting the in-depth understanding of the renal protective effect of empagliflozin. Additionally, due to the absence of blinding or placebo control in the study design, there is a possibility of subjective bias, which may affect the objectivity and reliability of the results. In terms of concomitant medication, there are certain differences in the use of antihypertensive, antidiabetic, and lipid-lowering drugs among the patients involved in the study, which may have an impact on the results and increase the variability of the study. Moreover, the limitation of sample size may also affect the generalizability and applicability of the results. Lastly, the patient selection in this study was limited to low-risk type 2 diabetes patients with normal albuminuria, which may not fully represent the entire population of type 2 diabetes patients. These limitations suggest that future research should adopt longer follow-up periods, larger sample sizes, designs with blinding or placebo control, and more comprehensive renal health indicators to more comprehensively and accurately evaluate the renal protective effect of SGLT2 inhibitors in type 2 diabetes patients. However, the innovation of this study lies in its incorporation of new perspectives on the pathogenesis of DKD and the evaluation of the role of SGLT2 inhibitors from the perspective of early renal tubular injury. This is the first study to include early-stage low-risk type 2 diabetes patients with normal albuminuria and evaluate the early renal tubular protective effect of SGLT2 inhibitors using multiple renal tubular injury markers.

**Fig. 10:**
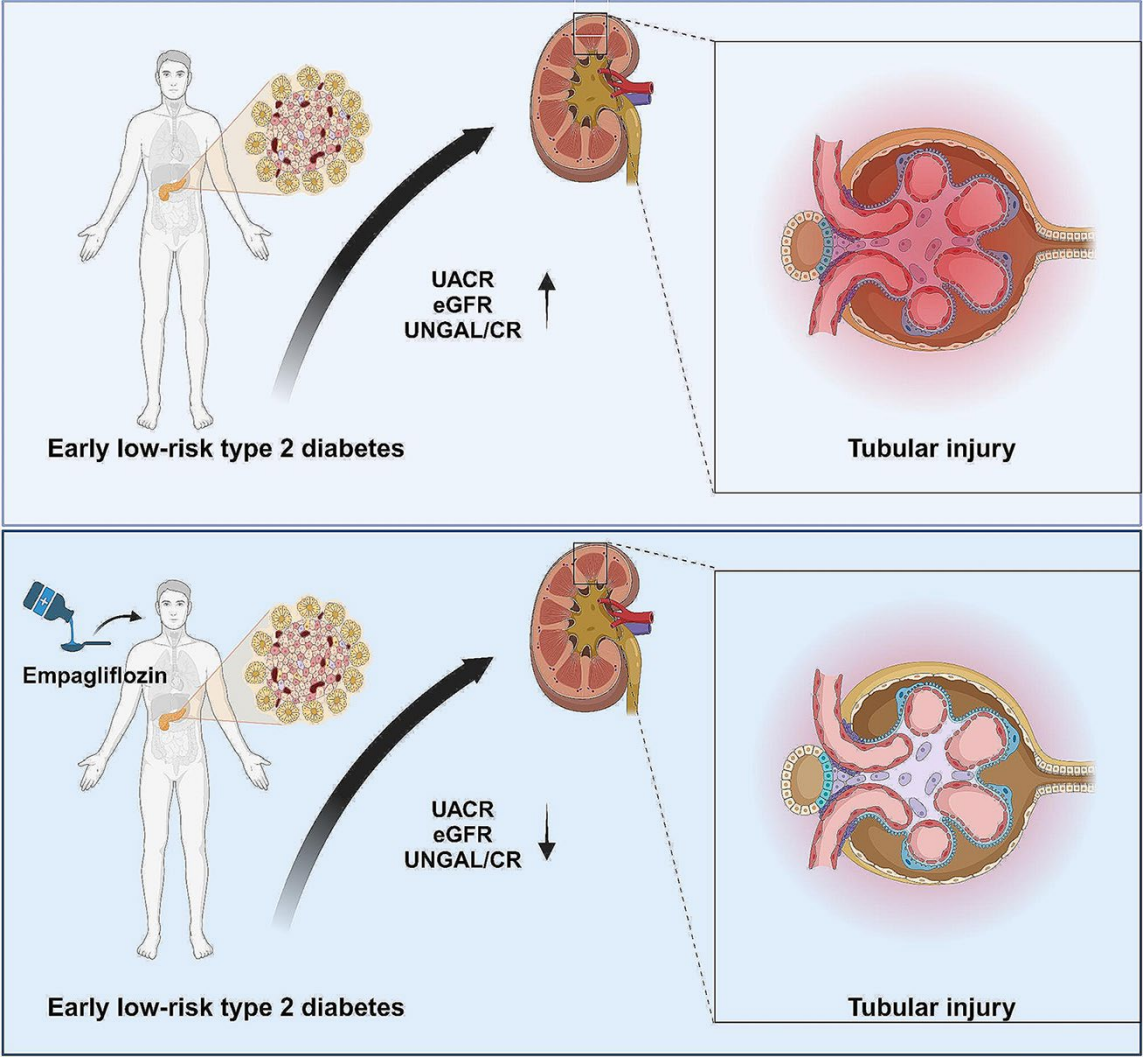
Illustration of the protective effect of empagliflozin on renal tubular injury in early-stage low-risk type 2 diabetes patients

Previous studies have reported that the SGLT2 inhibitor empagliflozin can improve diabetic kidney disease in type 2 diabetes patients. This study reveals a significant impact of empagliflozin, an SGLT2 inhibitor, in reducing the markers of tubular damage, KIM-1 and NGAL, in early-stage type 2 diabetes patients (Fig. 10). In comparison to prior research, our study is the first to report that empagliflozin can improve glomerular injury by lowering levels of KIM-1, A1M, B2M, and NGAL. The findings of this study demonstrate the dual regulatory effects of empagliflozin on blood glucose and lipid levels. In conclusion, this research provides novel therapeutic strategies and targets for renal protection in type 2 diabetic patients, further enhancing its significance as an integral part of comprehensive treatment for type 2 diabetes.

## Conclusion

For future research, longer follow-up periods are needed to observe the long-term renal protective effects of empagliflozin and its impact on overall cardiovascular health. Moreover, studies should include more renal health indicators and type 2 diabetes patients from different risk groups to comprehensively evaluate the effects of SGLT2 inhibitors. Exploring their mechanisms of action at the molecular and cellular levels, as well as their role in comprehensive diabetes management, will provide important guidance for future treatment strategies and clinical practices. Overall, this study provides a new perspective on early renal protection in type 2 diabetes patients and paves the way for future research and clinical applications.

### Electronic supplementary material

Below is the link to the electronic supplementary material.

Supplementary Material 1

## Data Availability

All data can be provided as needed.
