# Supplementary material for: Empagliflozin’s role in early tubular protection for type 2 diabetes patients
Source: Mol Med. 2024 Jul 31;30:112. doi: 10.1186/s10020-024-00881-0 (PMC11293177; doi:10.1186/s10020-024-00881-0)
Supplement: Supplementary file 1 — Supplementary Material 1 [file 10020_2024_881_MOESM1_ESM.docx]

Reviewer reports:

**Reviewer #1:** This randomized controlled clinical study evaluates the effect of SGL2 inhibitor (empagliflozin) on early tubular injury in patients with type-2 diabetes. It is a well-designed clinical study. However, the manuscript lacks mechanistic insights and details on dissecting the renoprotective role of SGL2 inhibitors evaluated in previous trials. This reviewer would request major revisions addressing the following comments:

1. Did the authors measure other sensitive and well-characterized early biomarkers of tubular injury? These include IL-18, L-FABP, A1M, B2M, etc. This will greatly add to the strength of this manuscript.

**Response:** Thank you for your suggestion. We have included this information in the text, with detailed specifics presented in Table 3. Following treatment, the markers IL-18, A1M, B2M, and L-FABP exhibited a decrease.

2. Are there any pre-clinical studies on mouse models of early tubular injury where empagliflozin has shown a protective role? Please cite these studies, mention details on mechanisms involved.

**Response:** During our search of preclinical studies, we found that Lu et al. demonstrated in a diabetes mouse model that Empagliflozin prevents ferroptosis by promoting the AMPK-mediated NRF 2 activation pathway, thereby alleviating renal tubular damage in diabetic mice (PMID: 36581059). The outcomes of this study further support the conclusions drawn in our paper, with corresponding explanations now included in the discussion section.

3. It is important to mention the average dose of other anti-diabetic medications given to the control group versus the intervention group. Depending on the dose, both oral hypoglycemic agents and insulin can have a dramatic effect on metabolic parameters other than blood glucose.

**Response:** We regret the oversight of not statistically analyzing the average types and doses of antidiabetic medication between the control and intervention groups. However, the primary medication used was metformin, with specific dose information now added to Table 1.

4. Were any these patients taking lipid-lowering agents other than statins? Please also include average dose of statins received by the control versus the intervention group in table 1.

**Response:** Apart from statins, no other lipid-lowering medications were administered. The average dosage of statins, the lipid-lowering drugs used in both the experimental and control groups, has been incorporated into the revised Table 1.

5. The arrow/curve X-Y graphs next to each bar graphs do not seem to add any value to the data already represented in the bar graphs and hence are recommended to be removed. The interaction effect can be mentioned simply below the bar graphs.

**Response:** We agree that the X-Y graphs may not add significant value and have decided to remove them, simplifying the presentation of our results as suggested.

6. Throughout the results, the authors compare before treatment vs after treatment for each group. While this is a good comparison, in order to emphasize effect of intervention (empagliflozin), it is important to statistically compare After treatment-control group versus After treatment-intervention group. Please highlight p-values for this comparison on the bar graphs. The renoprotective role on kidney function and metabolic parameters can only be claimed if after treatment values are significantly different between the control and intervention groups. Please revise the manuscript and claims accordingly.

**Response:** We have added P-values on the bar graphs in all figures to emphasize the comparative effectiveness between the two groups after Empagliflozin treatment, elucidating the protective effect of statin medication on the kidneys. Additionally, we have supplemented the legends of the figures to explain the significance of the symbols denoting differences.

Minor comment:

1. If the after treatment-HbA1c values are available - please plot in figure 2.S

**Response:** After treatment with Englitazone, there was also an improvement in the levels of HbA1c. The statistically adjusted changes in HbA1c levels have been incorporated into the revised Table 2 and Figure 2C, aiming to enhance the comprehensiveness of glycemic assessment parameters.

**Reviewer #2:** The manuscript by Zhang C et al. titled " Empagliflozin's Role in Early Tubular Protection for Type 2 Diabetes" presents findings from a randomized controlled clinical trial evaluating the impact of the SGLT2 inhibitor empagliflozin on early tubular injury biomarkers in type 2 diabetes patients with normal albuminuria. The study focused on assessing KIM-1 and NGAL from urine as early tubular injury biomarkers. The results indicated that empagliflozin treatment led to improvements in these biomarkers, suggesting a potential role in early renal protection for type 2 diabetes patients. However, there are some points should be addressed as following:

1 Page 1, please change the abstract into English

**Response:** We apologize for the oversight. The abstract has been translated into English to ensure it is accessible to all readers.

2 Table 2, since there is an improvement of fasting blood glucose level in treatment group and control group, what is the HbA1c level of these two group after treatment? Is there any improvement as well?

**Response:** Following Englitazone treatment, the levels of HbA1c showed improvement. We have included the statistically adjusted changes in HbA1c levels in the revised Table 2 and Figure 2C.

3 The authors used tubule injury markers KIM-1 and NGAL to assess the progression of DN. Does the expression of these markers corelated with the level of ACR or eGFR in these patients?

**Response:** When eGFR increases, it signifies an improvement in renal filtration function. Given that the decrease in KIM-1 and NGAL levels is typically associated with reduced renal damage, it can be inferred that the improvement in eGFR may be linked to the decrease in KIM-1 and NGAL levels. We utilized KIM-1 and NGAL to assess the progression of Diabetic Nephropathy in the study, which is correlated with the improvement in eGFR (PMID: 33951537). The relevant section discussed in the article has been revised accordingly.

4 Table 3, It seems like ACR in control group also decreased after treatment? How to explain this result?

**Response:** While the control group showed changes in ACR values before and after treatment, these changes did not reach statistical significance (P > 0.05). ACR values only reflect the ratio of urinary albumin to creatinine and do not fully represent overall kidney function. Renal function encompasses several aspects such as filtration, reabsorption, and secretion, whereas ACR values only reflect one of these aspects. Nonetheless, patients in both groups exhibited stabilized blood glucose levels during treatment, a phenomenon usually associated with a reduction in urinary albumin. In a hyperglycemic state, glucose may pass through damaged glomerular filtration membranes into the urine, resulting in positive urine protein. Controlling blood glucose improves the function of the glomerular filtration membranes, consequently reducing the production of urine protein. Therefore, the slight decrease in ACR may be attributed to the stable blood glucose levels in the patients.

5 Since there are so many trials already showed that Empagliflozin and other SGLT2 inhibitors could improve and protect tubule injury and DN progression, please discuss further what is the novelty and uniqueness of this study.

**Response:** Previous research has documented the ability of the SGLT2 inhibitor empagliflozin to improve diabetic nephropathy in type 2 diabetes (PMID: 37616058). This study reveals a significant impact of empagliflozin, an SGLT2 inhibitor, in reducing the markers of tubular damage, KIM-1, and NGAL in early-stage type 2 diabetes patients (Figure 10). In comparison to previous studies, our research is the first to report on empagliflozin's capacity to ameliorate glomerular damage by reducing KIM-1, A1M, B2M, and NGAL levels. The results of this study demonstrate empagliflozin's dual regulatory effects on blood glucose and lipid levels. Taken together, this study offers a new therapeutic strategy and target for the protection of the kidneys in type 2 diabetic patients.
